# Should Five Years' Practice in Dentistry, Inclusive of Pupilage, Continue to Be Regarded as Equivalent to One Course of Lectures in Dental Colleges?

**Published:** 1877-11

**Authors:** John H. M'Quillen

**Affiliations:** Professor of Physiology in Philadelphia Dental College.


### ARTICLE III.

### *Should Five Years' Practice in Dentistry, Inclusive of Pupilage, Continue to be Regarded as Equivalent to One Course of Lectures in Dental Colleges?*

BY JOHN H. M'QUILLEN, M. D., D. D. S.,
Professor of Physiology in Philadelphia Dental College.

*Read before the Pennsylvania State Dental Society, at the Ninth Annual Meeting, held July,* 1877.

The chairman of the Executive Committee wrote to me two months ago to prepare an essay for this meeting of the Society. I responded that it would not be convenient for me to do so. In looking, however, over the order of exercises, which has just come to hand (a few days before the meeting,) I observe that in the list of essays to be presented "Dental Education" is not named. Under these circumstances, with no intention of inflicting upon the members a lengthy and elaborate essay on that important theme, I have thought there might be some propriety in submitting a few suggestions, hastily written, under the query that heads this communication. Brief as the article is, if it should provoke discussion, awaken the minds of the profession to the impropriety of continuing to regard "five years' practice in dentistry, inclusive of pupilage, as equivalent to one course of lectures," and lead to its abandonment on the part of the dental colleges, my object will have been attained.

In the organization of the National, State and local dental societies, dental education was one of the primary and important objects in view. Recognized, indeed as the cornerstone of the foundation, on which an enduring superstructure could be erected, that would command the respect of those engaged in the liberal professions, the founders of these societies advocated the thorough education of those proposing to enter upon the study of dentistry. How that could be best accomplished, whether in departments connected with medical colleges, or in dental colleges, was then, and still continues to be, a prolific theme of discussion. With no intention of entering upon the consideration of these differences of opinion, it is an undeniable fact that the dental colleges have been the active agents in effecting the wonderful changes that have taken place in the profession, and in the estimate of its practitioners by the community within the past fifty years. From what was formerly in the hands of a few men—little more than a mere mechanical occupation—it has been elevated to the rank of a liberal profession; and the dentist, in place of being regarded as an object of fear and dread, whose delight it was to wrench from the jaw an aching tooth in the most unfeeling manner, is now looked upon with respect and gratitude as an educated gentleman, whose mission is to relieve suffering humanity of the most painful of all afflictions, arrest the progress and restore the ravages made by decay in the natural organs; and when these conservative efforts prove unavailing, by the introduction of properly-constructed artificial substitutes to give to the features their natural expression.

It may be said, and truly, that a large proportion of the profession have never been inside of dental colleges, and that the dental societies and magazines have been the most powerful levers in accomplishing this great work. With no disposition to undervalue these agencies—on the contrary, recognizing to the fullest extent the power of the press, and the stimulating and beneficial influence of associated effort—a careful examination will reveal the fact that the organization of the National, State and local societies, in nearly every instance, has been the work of the faculties and graduates of dental colleges, and that the editors of, and most frequent contributors to the dental journals, have been Professors in the dental colleges, who have hereby reached a larger circle of students than could have come within the sound of their voices, and thus have made their influence felt, not only in every section of this country, but in addition, in every quarter of the globe where dentistry is practiced.

Several years ago I directed attention to the fact that, "a little more than a quarter of a century ago (thirty-eight years) a successful effort was made to establish a college for the express purpose of teaching the science and art of dentistry, in a manner calculated to elevate it to the rank of a liberal profession. As year after year rolled by, efforts were made, with varying success, to found institutions of similar character in different sections of the country. Some of these, after an ephemeral and inglorious career, ceased to exist; but the greater number have increased in strength and usefulness. Institutions of learning, as a rule, like individuals and nations, are compelled to enter upon their career with limited means, and whatever may be the aspirations and desires of their founders—with the fullest possible recognition of all that is demanded for the successful accomplishment of the aims and objects in view—stern necessity compels them to accommodate themselves to circumstances and make the most of their surroundings, with the determination, as time and increase of resources afford the means and power, to do that which was impossible to effect in the commencement of the undertaking. As the seed laid in the graund, under favoring influences, germinates and produces a thousand fold, so with increase of years, strength and resources, evidences of growth should be manifested in collegiate institutions, by enlarging the curriculum of instruction, increasing the means of illustration, and in every direction affording the fullest opportunity possible for the impartation and acquisition of knowledge.

"The trials and difficulties with which the faculty of each institution has had to contend in gaining the confidence and support of the profession, cannot be appreciated by those who have not participated in the struggle from the beginning; nor can they conceive the more than missionary zeal that is demanded in delivering regular and systematic courses of lectures to classes so small that the prospect of ultimate success appears impossible to any other than enthusiasts. A distinguished writer has said, 'It is pleasing to contemplate a manufacture rising gradually from its first mean state by the successive labor of innumerable minds; to consider the first hollow trunk of an oak, in which, perhaps the shepherd could scarce venture to cross a brook, swelled with a shower, enlarged at last into a ship of war, attacking fortresses, terrifying nations, setting rooms and billows at defiance, and visiting the remotest parts of the globe. And it might contribute to dispose us to a kinder regard for the labors of one another, if we were to consider from what unpromising beginnings the most useful productions of art have probably arisen. Who, when he saw the first sands of ashes, by a casual intensity of heat, melted into a metalline form, rugged with excrescences and clouded with impurities would have imagined that in this shapeless lump lay concealed so many conveniences of life as would in time constitute a great part of the happiness of the world? Yet by some such fortuitous liquefaction was mankind taught to procure a body at once in a high degree solid and transparent, which might admit the light of the sun and exclude the violence of the wind; which might extend the sight of the philosopher to new ranges of existence, and charm him at one time with the unbounded extent of the material creation, and at another with the endless subordination of animal life, and what is yet of more importance, to supply the decays of nature and succor old age with subsidiary sight. Thus was the first artificer in glass employed, though, without his own knowledge and expectation, he was facilitating and prolonging the enjoyment of light, enlarging the avenues of science, and conferring the highest and most lasting pleasures; he was enabling the student to contemplate nature, and the beauty to behold herself.'

"The origin and establishment of dental colleges have been so recent that ample opportunities have been afforded, not only to the profession, but the public at large, to observe their gradual development by the successive labors of a few active, energetic and indefatigable minds; and whatever may have been the doubts in the past, the question of the success of the movement is no longer an uncertain one."

These efforts have been subjected to constant criticism on the part of the profession, particularly at the meetings of the Associations. Some of these criticisms have been just, while others have been not only unjust, but ungenerous, and even willful perversion of facts. It is not only my intention to dwell on all the points that might be considered, but to direct attention to the fact that leading members of the profession have over and again urged, that the dental colleges should require of all students of dentistry attendance upon at least two full winter courses of lectures. And I would now ask the representatives of the profession in this and other State Dental Societies, and in the American Dental Association, whether you are in earnest in this matter, and are willing to prove it by acts as well as words ? You have the power. It is but for you to suggest, and the dental colleges would promptly act. Your request would be regarded in the light of a command.

When dental colleges were first established, the custom of regarding "five years' practice in dentistry, inclusive of pupilage, as equivalent to one course of lectures" was inaugurated, and found its justification in the fact that there were those who had been engaged in practice years before the foundation of such institutions, whose knowledge and skill was quite equal, if not superior, in some instances, to those of the professors, and therefore warranted the exception in their favor. Year after year this exception has been continued, and many excellent men, *of studious habits*, who have graduated under this clause, are an honor to the profession. There are still some estimable practitioners entitled to its provisions who have failed to take advantage of it, and in whose cases exceptions might be made in favor of fifteen or twenty years' reputable practice. But to continue to hold out the inducement that "five years' practice in dentistry, inclusive of pupilage, will be regarded as equivalent to one course of lectures," can be defended on no other ground than that of expediency, in securing a certain class of students, while at the same time, it is a standing advertisement in the college announcements, encouraging and indeed offering a premium to young men to enter the offices of practitioners, who are willing to take them, with the understanding that after remaining a few months, and in many instances but a few weeks, they can engage in practice on their own account, and with such inadequate preparation, after doing all the injury they can to the community and the profession, subsequently taking advantage of the "five years'" clause in the college announcement, graduate in one winter course of lectures. If this clause was rescinded by all the colleges, these men would be forced to attend at least two full winter courses, and thus undergo a training that would fit them to engage in practice with some credit to themselves and benefit to the community in which they may settle. As I have elsewhere asked, "What are the practitioners of dentistry doing individually with regard to the education of the profession? Is it not true that a man can enter the majority of dental offices, and after a short period—a few weeks or months at best, during which he has had few, if any opportunities beyond those afforded in the laboratory of learning anything about dentistry—engage at once in practice, with a certificate from his preceptor endorsing his competency? Are those who so freely criticise dental colleges doing their part as private preceptors? Do they inquire into the mental, moral and physical qualities of those whom they receive as private students; supply them with the latest and best text-book; direct their course of reading, and subject them to stated examinations, to ascertain what progress they may have formed? A few members of the profession do this, but they are exceedingly limited in number, and the yearly additions to the profession are mainly through private preceptors, who fail to do their duty by those who come under them as students; only a small proportion of the latter attend college and graduate. The alumni of the dental colleges have done much toward the advancement of the profession during the past few years, and they embrace among their number some of the most cultivated minds and skillful practitioners in our ranks. For years I have advocated the enactment of State laws which would make it obligatory upon students of dentistry that they should undergo two years of private preceptorship, attend two full courses of lectures, and graduate from a respectable dental college, before entering upon practice.

As one illustration out of many which might be cited of the manner in which private preceptors, so-called, at home and abroad, fail to discharge their duty to students, permit me to cite the following case. This spring a gentleman wrote to me in relation to a young man who had been engaged in dentistry for five years—four years in Europe, and one in America—and desired to graduate at the Philadelphia Dental College by attending one course of lectures. To this I replied, as in a number of similar applications, that unless the young man had attended a winter course of lectures in some other dental or medical college, it would be necessary for him to attend two full winter courses of lectures to entitle him to present himself as a candidate for graduation, as the faculty had rescinded the clause that "five years' practice in dentistry, inclusive of pupilage, will be regarded as equivalent to one course of lectures." In reply to this, a letter was received stating that this young man would start for Philadelphia immediately. He arrived in a few days and presented the following letter:

"DEAR SIR: I have the honor to introduce to you herêby, Mr. ——————, who is to enter your college, and whom I take the liberty of recommending to your special care."

In the interview with the young man, he gave me to understand that it was absolutely necessary for him to graduate at the expiration of the next winter course, as he had to return to his native country at that time to perform military duty. On asking what he had done during the past five years, he said his time had been passed in the laboratories of various dentists in Germany whom he named, and with one in this country who had given him some opportunities to operate. He had never studied anatomy, physiology or chemistry. Putting a few leading questions to him, which a young school boy would have answered, and he could not, I then endeavored to impress upon his mind the importance and extent of these studies; how utterly hopeless would be the task of undertaking to master a knowledge of them in the brief time he had named; and if he were ever so skillful as an operator and as a mechanical dentist—a fact yet to be proved by actual demonstration—I did not see how any college could regard *his* five years in dentistry as equivalent to one course of lectures, as it would be a very free interpretation of the clause to accept him as coming within its provisions, as he had never been *a student* or in actual practice on his own account, and merely occupied the position of a workman for others. Five years' work at the jewelers' bench, in a machine shop, or with a jack plane of the carpenter, might with equal propriety be regarded as an equivalent. That over again, in former years, I had declined to receive persons like himself as coming within the clause, and had induced them to attend two full winter courses of lectures; and that we could not receive him as eligible to graduate on one course, as we required attendance upon two full winter courses, which he was advised to take advantage of. He replied that he could not do so, and would have to go where he could make such arrangements as he had named. The young man spoke very good English. I name this, as it might be inferred that his inability to answer the questions was due to a want of familiarity with our language. The questions were plain and simple ones, with the view of ascertaining whether he had any knowledge of the subjects named. Some idea of their nature may be gained by one of them, viz., How many cavities are there in the human heart? Answer: Two. The young man was by no means deficient in intelligence. On the contrary he impressed me that by taking sufficient time to properly study the sciences named, he would have no difficulty in mastering them. The interview was a pleasant and friendly one throughout, and the young gentleman subsequently called and thanked me for the courtesy and attention which he had received.

At a period when many young men who have enjoyed every advantage in education not only attend two full winter courses of lectures, but in addition take two spring and fall courses, and work daily in the dispensary and laboratory of the Dental College for two years, with the view of properly fitting themselves for the practice of dentistry, it is an act of injustice to them, and one which they may very properly complain of, when discrimination is made in favor of men who have had none of their advantages, and yet are permitted to graduate on one term.

In conclusion, every one must admit that the interests of the profession, the community, the colleges, and last, though not least, the students, alike demand the rescinding of the clause that "certified evidence of having had five years' practice in dentistry, inclusive of pupilage, will be regarded as eqivalent to one course of lectures," and that in place thereof attendance on two full winter courses of lectures should be required of candidates for graduation by every respectable Dental College.

That the motives in writing this communication may be mis represented by interested parties, is naturally to be expected; but the principles on which it is based, cannot be controverted, or overturned.

